# Supplementary material for: System identification and surrogate data analyses imply approximate Gaussianity and non-stationarity of resting-brain dynamics
Source: bioRxiv. 2026 Mar 28:2026.03.25.714361. Preprint. [Version 1] doi: 10.64898/2026.03.25.714361 (PMC13041960; doi:10.64898/2026.03.25.714361)
Supplement: Supplement 1 [file NIHPP2026.03.25.714361v1-supplement-1.pdf]

bioRxiv preprint doi: https://doi.org/10.64898/2026.03.25.714361; this version posted March 28, 2026. The copyright holder for this preprint (which was not certified by peer review) is the author/funder, who has granted bioRxiv a license to display the preprint in perpetuity. It is made available under aCC-BY 4.0 International license.

Supplementary Figures

bioRxiv preprint doi: https://doi.org/10.64898/2026.03.25.714361; this version posted March 28, 2026. The copyright holder for this preprint (which was not certified by peer review) is the author/funder, who has granted bioRxiv a license to display the preprint in perpetuity. It is made available under aCC-BY 4.0 International license.

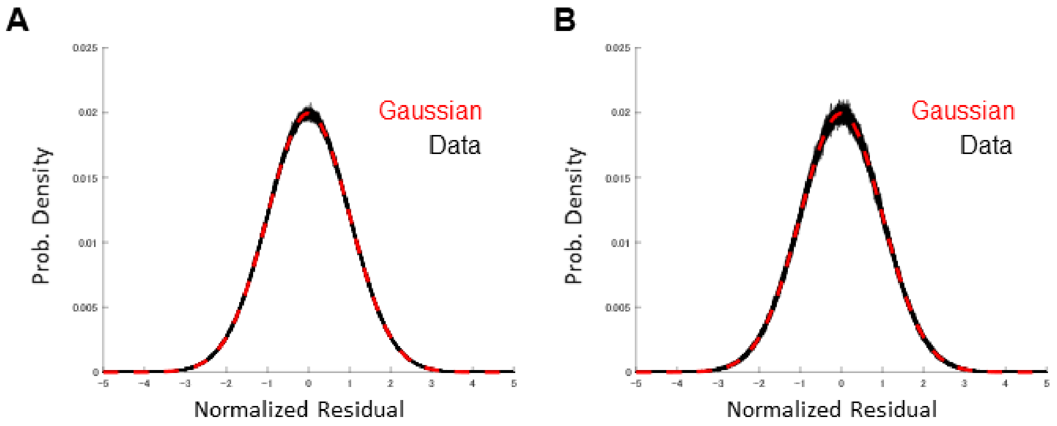

Figure S1. Same as Figure 2F and 4F but for PR surrogates. (A) Same plot as Figure 2F, but for PR surrogates of scan-concatenated data. **(B)** Same plot as Figure 4F, but for PR surrogates of single-scan data.

bioRxiv preprint doi: https://doi.org/10.64898/2026.03.25.714361; this version posted March 28, 2026. The copyright holder for this preprint (which was not certified by peer review) is the author/funder, who has granted bioRxiv a license to display the preprint in perpetuity. It is made available under aCC-BY 4.0 International license.

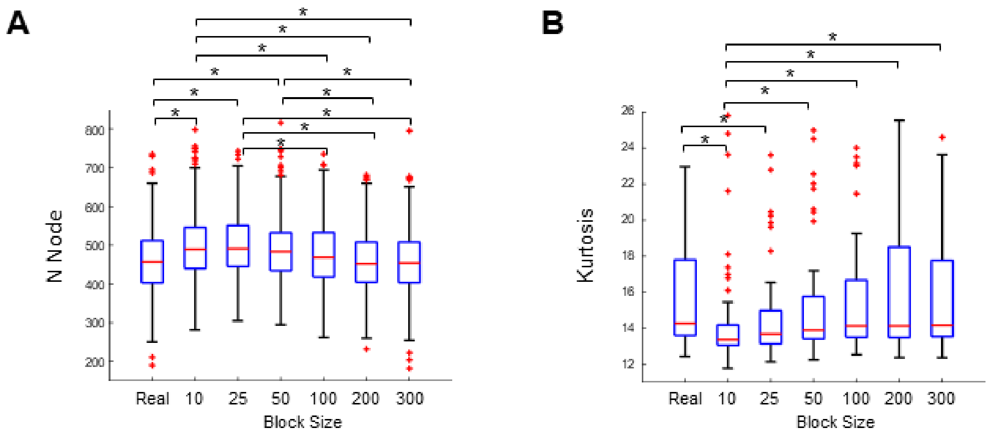

Figure S2. Same as Figure 5C and E but significant differences are indicated. (A) Same plot as Figure 5C, but significant differences are indicated. **(B)** Same plot as Figure 5E, but significant differences are indicated. (i.e., excluding interpolated frames). *, $p < 0.05$ (Dunn's test, Bonferroni corrected).

bioRxiv preprint doi: https://doi.org/10.64898/2026.03.25.714361; this version posted March 28, 2026. The copyright holder for this preprint (which was not certified by peer review) is the author/funder, who has granted bioRxiv a license to display the preprint in perpetuity. It is made available under aCC-BY 4.0 International license.

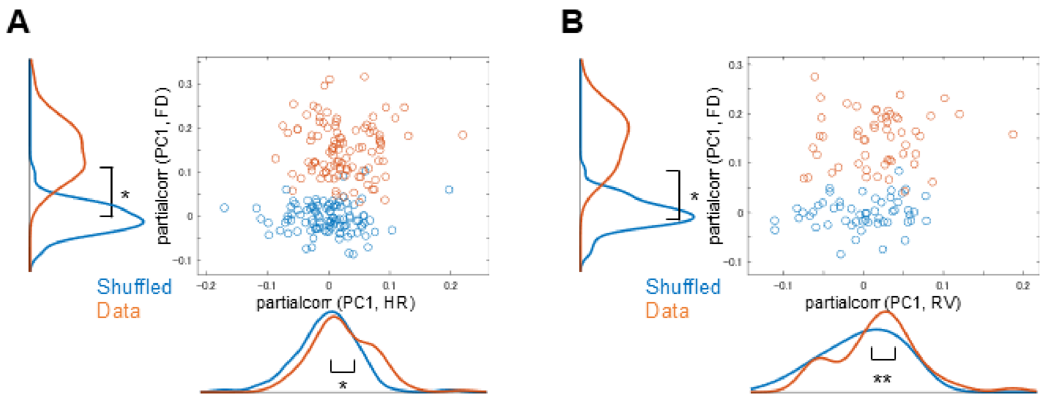

Figure S3. Same as Figure 6B and C but calculated using only censored frames. (A) Scatter plot showing correlation between PC1 and HR (FD) partialized for FD (HR) for all scans selected for good HR measurement (104 scans). Same convention as Figure 6B, calculated using only censored frames (i.e., excluding interpolated frames). **(B)** Scatter plot showing the correlation between PC1 and RV (FD) partialized for FD (HR) for all scans selected for good RV measurement (51 scans). Same convention as Figure 6C, calculated using only censored frames (i.e., excluding interpolated frames). *, $p < 0.001$, **, $p < 0.05$ (two-sample t test).
